# Representation of CYP3A4, CYP3A5 and UGT1A4 Polymorphisms within Croatian Breast Cancer Patients’ Population

**DOI:** 10.3390/ijerph17103692

**Published:** 2020-05-23

**Authors:** Kristina Bojanic, Lucija Kuna, Ines Bilic Curcic, Jasenka Wagner, Robert Smolic, Kristina Kralik, Tomislav Kizivat, Gordana Ivanac, Aleksandar Vcev, George Y. Wu, Martina Smolic

**Affiliations:** 1 Department of Biophysics and Radiology, Faculty of Dental Medicine and Health Osijek, J. J. Strossmayer University of Osijek, 31000 Osijek, Croatia; kristina.bojanic@dzo.hr; 2 Department of Biophysics and Radiology, Faculty of Medicine Osijek, J. J. Strossmayer University of Osijek, 31000 Osijek, Croatia; 3 Department of Radiology, Health Center Osijek, 31000 Osijek, Croatia; 4 Department of Pharmacology and Biochemistry, Faculty of Dental Medicine and Health Osijek, J. J. Strossmayer University of Osijek, 31000 Osijek, Croatia; lucija.kuna@fdmz.hr; 5 Department of Pharmacology, Faculty of Medicine Osijek, J. J. Strossmayer University of Osijek, 31000 Osijek, Croatia; ibcurcic@mefos.hr; 6 Department of Medicine, Division of Endocrinology, University Hospital Osijek, 31000 Osijek, Croatia; 7 Department of Medical Biology and Genetics, Faculty of Medicine Osijek, J. J. Strossmayer University of Osijek, 31000 Osijek, Croatia; jwagner@mefos.hr; 8 Department of Pathophysiology, Physiology and Immunology, Faculty of Dental Medicine and Health Osijek, J. J. Strossmayer University of Osijek, 31000 Osijek, Croatia; rsmolic@mefos.hr (R.S.); avcev@fdmz.hr (A.V.); 9 Department of Pathophysiology, Faculty of Medicine Osijek, J. J. Strossmayer University of Osijek, 31000 Osijek, Croatia; 10 Department of Medicine, Division of Gastroenterology/Hepatology, University Hospital Osijek, 31000 Osijek, Croatia; 11 Department of Medical Statistics and Medical Informatics, Faculty of Medicine Osijek, J. J. Strossmayer University of Osijek, 31000 Osijek, Croatia; kristina.kralik@gmail.com; 12 Clinical Institute for Nuclear Medicine and Radiation Protection, University Hospital Osijek, 31000 Osijek, Croatia; tomislavkizivat@gmail.com; 13 Faculty of Medicine Osijek, J. J. Strossmayer University of Osijek, 31000 Osijek, Croatia; 14 Department of Diagnostic and Interventional Radiology, University Hospital Dubrava, 10000 Zagreb, Croatia; gordana.augustan@gmail.com; 15 University of Zagreb School of Medicine, Salata 3, 10000 Zagreb, Croatia; 16 Department of Medicine, Division of Gastrenterology/Hepatology, University of Connecticut Health Center, 263 Farmington Avenue, Farmington, CT 06032, USA; wu@uchc.edu

**Keywords:** single nucleotide polymorphism, drug metabolizing enzyme, breast cancer therapy, anastrozole, side effects

## Abstract

Single nucleotide polymorphism (SNP) in genes encoding drug-metabolizing enzymes (DME) could have a critical role in individual responses to anastrozole. Frequency of CYP3A4*1B, CYP3A5*3 and UGT1A4*2 SNPs in 126 Croatian breast cancer (BC) patients and possible association with anastrozole-induced undesirable side effects were analyzed. Eighty-two postmenopausal patients with estrogen receptor (ER)-positive BC treated with anastrozole and 44 postmenopausal ER-positive BC patients before hormonal adjuvant therapy were included in the study. Genomic DNA was genotyped by TaqMan Real-Time PCR. Bone mineral density (BMD) was measured by dual-energy X-ray absorptiometry. The homozygotes for the variant G allele of CYP3A5*3 were predominant (88%), and the homozygotes for the reference A allele were not detected. While homozygotes for the variant G allele of CYP3A4*1B were not detected, predominantly wild type homozygotes for A allele (94%) were present. CYP3A4*1B and CYP3A5*3 SNPs were in 84.3% linkage disequilibrium (D’ = 0.843) and 95.1% (D’ = 0.951) in group treated with anastrozole and w/o treatment, respectively. Homozygotes for the A allele of UGT1A4*2 were not detected in our study groups. Although the variant CYP3A5*3 allele, which might result in poor metabolizer phenotype and more pronounced side effects, was predominant, significant association with BMD changes induced by anastrozole were not confirmed.

## 1. Introduction

Breast cancer (BC) is the most common cancer site with 26% of all cancer sites in females. Two-thirds of all BC are estrogen receptor (ER) positive and, thus, are candidates for hormonal therapy [1]. Third generation aromatase inhibitors (AIs) such as anastrozole, letrozole and exemestane have demonstrated a longer disease-free survival, and better overall safety in the adjuvant treatment of postmenopausal women with ER-positive BC, compared with the selective ER modulator (SERM) Tamoxifen (TAM) [2,3]. The use of AIs as a first-line therapy or as a second-line therapy after treatment with Tamoxifen (TAM) is recommended. Although anastrozole was determined superior as and more effective than SERMs [4], some patients still experience cancer recurrence [5]. In addition, considerable interindividual variability in its tolerability was identified, which may lead to adverse effects [6,7,8]. This variability can potentially be attributed to individual differences in the pharmacokinetics and/or pharmacodynamics of anastrozole, possibly due genetic variations [9]. Anastrozole is primarily metabolized in the liver [5]. In phase I it undergoes oxidation predominantly by CYP3A4 and to a lesser extent by CYP3A5 to form hydroxyanastrozole, which may further be glucuronidated in phase II to glucuronid hydroxanastrozole by UGT1A4 [10]. It can also be directly glucuronidated to anastrozole N-glucuronide, predominantly catalyzed by UGT1A4 [10,11]. Currently, at least 78 nucleotide sequence variations of CYP3A4 have been identified and one of the most common variants is in the 5′promoter region (−290), marked as CYP3A4*1B (rs2740574) [12,13,14]. In comparison with the wild type CYP3A4*1A, CYP3A4*1B shows about a 2-fold increase in enzyme activity [15,16]. However, these data are not uniform, since a decrease in the catalytic activity of the enzyme was also determined [17], or unclear clinical effect was described [18], and some even linked this SNP with increased cancer risk [19,20]. Frequent single nucleotide polymorphism (SNP) of CYP3A5 gene is a substitution of adenine with guanine in intron 3: 6896A>G (rs776746). The mutated allele CYP3A5*3 results in a splicing defect of the mRNA and produces an unstable and nonfunctional protein with decreased activity, or loss of activity of the encoding enzyme, which according to some studies could play a role not only in interindividual variations but also in interethnic variations in anastrozole metabolism [21]. The mutated allele and the wild type were named CYP3A5*3 (rs776746) and CYP3A5*1, respectively. CYP3A4 and CYP3A5 polymorphisms frequencies also differ remarkably among different human populations [16,22]. The allelic frequency of CYP3A4*1B in Caucasians and African Americans is around 4%–9% and 59%–79%, respectively [21,23]. CYP3A5 is expressed differently among several ethnic groups, with CYP3A5*3 allele frequencies of 30%, 50% and 90% in African, Asian and Caucasian, respectively [24,25]. It was reported that the contribution of CYP3A5 to drug metabolism varies from 6% to 99% of the total CYP3A activity in different populations [25]. However, Kamdem, in his 2010 study, concluded that the role of any SNP in the CYP3A5 gene in vivo is probably small (because the in vitro kinetic data suggest CYP3A4 (not CYP3A5) is the dominant enzyme responsible for anastrozole metabolism at therapeutically relevant concentrations) [10]. Other studies suggest that it is difficult to differentiate the net contribution of CYP3A4*1B and CYP3A5*1, considering substantial overlap in CYP3A substrate specificity [20,25,26]. Interestingly, previous studies have shown significant linkage disequilibrium between CYP3A4*1A and CYP3A5*3 in Caucasians, and between CYP3A4*1B and CYP3A5*1 in African Americans [20,27].

UGT1A4 is the main enzyme responsible for direct glucuronidation of anastrozole in vitro, although the exact part of this enzyme to the in vivo metabolism of the drug is not yet fully known [10]. The UGT1A4 (UDP glucuronosyltransferase family 1 member A4) gene is highly polymorphic [28]. Glucuronidation activity in various human tissues shows a high degree of interindividual variability that cannot be explained by the unique tissue-specific expression of UGT1A4 gene [29,30]. Potential explanations of this variability are SNPs situated in the promoter and coding regions of the UGT1A4 gene, resulting in quantitative or qualitative changes in specific enzyme activity among individuals. In the UGT1A4 gene, replacement of cytosine with adenine in exon 1 was identified, that in vitro showed the altered (reduced) glucuronidation activity [31,32,33]. After in vitro studies, studies on the influence of this genetic variant on biotransformation of anastrozole in vivo were continued. However, according to studies by Lazarus and Sun and Kamdema et al., mutated alleles UGT1A4*2 (rs6755571) show no difference in glucuronidation activity compared to wild type [11,24], Ehmer’s research associates SNP with reduced glucuronidation activity against mutagenic amines and endogenous steroids, altering the liver metabolism of anastrozole and detoxification [28,34].

SNP in the genes encoding drug-metabolizing enzymes (DME) could have a critical role in individual responses to anastrozole. Even though these polymorphisms are well characterized in different populations, the frequency of CYP3A4*1B, CYP3A5*3 and UGT1A4*2 allele yet have not been determined in the population of Croatia (CRO), therefore the objectives of this study were to analyze the frequency of these SNPs and its possible association with anastrozole therapy-induced undesirable side effects among Croatian BC population.

## 2. Patients and Methods

### 2.1. Patients

A total of 126 subjects were included in the study consisting of 82 postmenopausal patients with ER-positive BC treated with anastrozole (mean age 65 (60–71.8)) and 44 newly diagnosed postmenopausal ER-positive BC patients before hormonal adjuvant therapy (mean age 62 (56–69)). The patients suffering from BC were collected at the Department of Ultrasound Diagnostics and Mammography at the Health Center Osijek. We randomly included all patients willing to participate with pathohistological BC subtype luminal A and luminal B at TNM stage I and II, i.e., without distant metastases. Patients treated with anastrozole were on hormonal adjuvant therapy at least one year up to a total of five years at the time of enrollment in the study. Ethical approval for this study was obtained from the Health Center Osijek Review Board, Ethics Committee for Research of the University, J.J. Strossmayer, Faculty of Medicine Osijek and Ethical Committee of Clinical Hospital Center in Osijek (approval number: 08-1621-1/15, 2158-61-07-15-128, R2-1099-4/2016). All research involving human subjects and material derived from human subjects in this study was done in accordance with ethical principles outlined in the World Medical Association Declaration of Helsinki—Ethical Principles for Medical Research Involving Human Subjects (initiated in June 1964, last amendment in October 2000). All participants signed an informed consent form before being included in the study. Subjects were excluded if they had a history of prior osteoporosis treatment or if they received treatment with medications that interfered with bone metabolism, such as chronic corticosteroids (>3 months duration). They were also excluded if they had a history of an eating disorder, primary hyperparathyroidism, untreated hyperthyroidism, or chronic kidney, gastrointestinal or liver disease. Blood samples were obtained from all participants in fasting conditions between 8 a.m. and 10 a.m. and were stored at −80 °C until assayed. In the group of anastrozole treated patients, sampling was done after including the patients in the study, that is, after at least one year of using anastrozole.

### 2.2. Methods

#### 2.2.1. Genotyping Analysis

Genetic variants were genotyped into three genes involved in anastrozole metabolism CYP3A4, CYP3A5 and UGT1A4, one polymorphism from each gene (rs2740574, rs776746 and rs6755571).

Genomic DNA was isolated from peripheral blood samples using a commercially available kit (QIAamp DNA Blood Midi Kit, Qiagen, Hilden, Germany) according to manufacturer’s protocol. Genotyping was performed on a 7500 Real-Time PCR system (Applied Biosystems, Foster City, CA, USA) using TaqMan Genotyping Master Mix Assay (Applied Biosystems, Foster City, CA, USA). Analysis of the allelic discrimination was performed with SDS 7500 Software Version 2.3 (Applied Biosystems, Foster City, CA, USA).

Location and proposed effects of CYP3A4*1B, CYP3A5*3 and UGT1A4*2 polymorphisms are summarized in Table 1.

#### 2.2.2. Assessment of Bone Mineral Density

Bone mineral density (BMD; g/cm^2^) was measured by dual-energy X-ray absorptiometry (DXA) imaging (Lunar Prodigy, GE Healthcare, SAD) at the lumbar spine, total hip and femoral neck, as described previously [32].

Lumbar spine BMD was determined using the anteroposterior projection and was calculated as the average of L1–L4. Both hips and proximal femur scans were used and mean values were calculated. In the AI group, BMD was measured after one year of AI treatment was initiated, while in the group w/o AI BMD was measured prior to anastrozole treatment. MD values were standardized as T-scores by the same operator according to the operating procedures of the manufacturer. The performance characteristics and standardized quality control procedures of these methods were described earlier [35,36].

#### 2.2.3. Statistical Analysis

Categorical data were represented by absolute and relative frequencies. The normality of the distribution of numerical variables was tested by the Shapiro–Wilk test. Numerical data were described by median and interquartile range. The Mann–Whitney U test was used to compare the median between two groups. Categorical variables were compared by Fisher’s exact test. The χ2-test was used to determine if the allele and genotype frequencies of polymorphisms fit the Hardy–Weinberg equilibrium. The linkage disequilibrium (LD) between specific CYP polymorphisms was calculated by the LD program. All *p*-values were two-sided. The level of significance was set at an alpha of 0.05. The statistical analysis was performed using MedCalc Statistical Software version 18.2.1 (MedCalc Software bvba, Ostend, Belgium; http://www.medcalc.org; 2018) and the IBM SPSS Statistics 23 (IBM Corp. Released 2015. IBM SPSS Statistics for Windows, Version 23.0. Armonk, NY: IBM Corp.).

## 3. Results

The study included 126 postmenopausal women diagnosed with ER-positive BC. Since initial DXA was not done prior to anastrozole introduction to all patients in the AI therapy group (health system limitations in Croatia), a control group of patients with newly discovered ER-positive BC prior to the hormone adjuvant therapy was formed in a way that we assigned a control to the subjects in the AI group with respect to demographic, anthropometric and other studied parameters.

There were no significant differences between the two groups in their height, height in youth, weight and BMI, as shown in Table 2. There were no significant differences between the two groups regarding lifestyle and eating habits, such as alcohol consumption, smoking, calcium intake and consumption of dairy products. Patients in the AIs treated group had a higher vitamin D intake. Women in group w/o AIs were more concerned with physical activity at the time research was conducted, as well as earlier in youth, and the majority of women exercised. There were no significant differences in the age of the first menstrual period, the age of menopause between the groups, and a large majority of participants had regular menstrual cycles throughout their lives (88.9%) (Table 2).

The CYP3A4*1B, CYP3A5*3 and UGT1A4*2 genotype frequencies were in line with Hardy–Weinberg equilibrium (*p* > 0.05). The genotype and variant allele frequencies for the CYP3A4*1B, CYP3A5*3 and UGT1A4*2 polymorphisms in the postmenopausal ER-positive BC patients from CRO are shown in Table 3.

As shown in Table 3, the homozygotes for the G allele CYP3A4*1B were not detected in our study groups. Homozygotes with reference A allele of CYP3A4*1A were predominantly present (94%). Six patients on anastrozole and one patient prior hormonal therapy were heterozygotes for CYP3A4*1B. The frequency distribution for CYP3A5*3 showed that homozygotes for the variant G allele were most abundant (88%). Homozygotes for the A allele of CYP3A5*1A were not found in BC patients treated with anastrozole, nor in the group w/o therapy. All six subjects who were heterozygous for CYP3A4*1B were also heterozygous for CYP3A5*3 allele. Thus, CYP3A4*1B and CYP3A5*3 polymorphisms were in 84.3% linkage disequilibrium (D’ = 0.843) in our study group treated with anastrozole, and 95.1% in the group w/o (D’ = 0.951).

Regarding UGT1A4*2, homozygotes for the for the variant A allele were not detected in our study groups. Three patients were heterozygotes for mutant allele of UGT1A4. Homozygotes for the wild C allele were predominantly present (96%).

We also analyzed the possible association of undesirable side effects induced by anastrozole therapy with the prevalence of CYP3A4*1B, CYP3A5*3 and UGT1A4*2 polymorphisms. However, no significant association in allele frequencies for CYP3A4*1B, CYP3A5*3 and UGT1A4*2 polymorphisms with changes in BMD was demonstrated between patients on anastrozole therapy and the ones prior therapy in our study (Table 4 and Table 5).

## 4. Discussion

Pharmacogenetics studies showed that polymorphisms of DME, transporters and receptors contribute to variable drug response [37]. CYP3A4 and CYP3A5 isoenzymes metabolize more than 50% of all prescription drugs, such as cholesterol-lowering drugs, calcium channel antagonists, immunosuppressants, antibiotics, oral anticoagulants and anticancer chemotherapeutic drugs [23]. Therefore, polymorphisms in the CYP family may be an important genetic contributor to interindividual and interracial differences in CYP3A-dependent drug clearance and response like that for the anastrozole. In addition, assessment of CYP3A4, CYP3A5 and UGT1A4 variant alleles and knowledge about their allelic frequency in the Croatian population may lead to personalized breast cancer therapy.

Studies demonstrated that frequency of CYP3A4*1B and CYP3A5*3 polymorphisms differ among ethnic groups [21,23,38]. Our results confirmed that frequencies of CYP3A4*1B and CYP3A5*3 alleles in Croatian subjects were in line with the Caucasians genotype data reported earlier [18]. We proved that CYP3A4*1B allele is rare in Croatian BC patients population, with heterozygotes CYP3A4*1A/CYP3A4*1B being present in only 6%, while CYP3A4*1B/CYP3A4*1B homozygotes were not detected. Similarly, previous studies reported frequency of CYP3A4*1B allele in about 4%–9% Caucasians, which is much lower than in African Americans with the frequency of this allele around 53% [21,38], but being higher relative to Taiwanese and Chinese with frequency of 0% [39]. In contrary, CYP3A5*3 polymorphism was found to be predominant in the Croatian population with an incidence of 88.1%. These data are in accordance with previous studies performed in other European populations, such as 94.9% in the Bosnia and Herzegovina population as the first neighboring country [22], and 94.35% in the Greek [23], 94% British [40], 91.7% Dutch [26], 87.5% Portuguese [21] and 82% French populations [41].

Furthermore, here we have identified a simultaneous existence of specific genotypes of CYP3A4*1B and CYP3A5*3 in the same BC patient. All six subjects with CYP3A4*1B allele were also defective for CYP3A5*3 allele. Thus, our data complement data from previous studies, which indicated a strong linkage disequilibrium between mutant CYP3A5 and CYP3A4 alleles in Caucasians [22,27].

Our results for UGT1A4*2 are not straightforward since they differ from results in study by Ehmer, who reported minor allele frequency of UGT1A4*2 (P24T) variant of 8% [28]. Ehmer et al. concluded that the high prevalence of SNPs throughout the human UGT1A gene locus is a genetic basis of interindividual variations of hepatic metabolism. UGT1A4*2 polymorphism of the hepatic UGT1A4 protein shows a differential metabolic activity toward mutagenic amines and endogenous steroids, altering hepatic metabolism and detoxification [28]. These results were confirmed in the study by Zhou, who demonstrated decreased function of UGT1A4*2 on lamotrigine glucuronidation in vitro, which may lead to interindividual variations in lamotrigine metabolism in vivo [33]. Our results about the prevalence of UGT1A4*2 alleles in Croatian BC patients could primarily be the result of a small sample size, however, the effect of socioeconomic factors cannot be excluded. In the study by Jean-Nicholas Roy on the CYP3A5 genetic variant, however, it was indicated that differences in SNPs frequencies may reflect evolutionary pressures generated by environmental factors in geographically distinct regions [42]. Data from literature suggest the complexity and interethnic variability of the UGT1A gene locus [43]. Further studies, including a larger number of subjects, primarily healthy women, will be necessary. Apart from limited sample size, a possible limitation of our research includes the inability to measure the concentration of anastrozole metabolite in serum at the time of the study. This drawback at the same time is precisely the potential of a new study to measure the concentration of metabolites of anastrozole, as well as estrogen, testosterone and androstendion concentrations in the serum, in addition to the research parameters included in the study so far. Up to now, only one such study was described in the literature by Ingle and his associates. They found that out of 191 patients with early breast cancer, two patients were extensively metabolizing anastrozole and did not show significant reductions in estrogen serum levels. We can assume that in such patients the anastrozole effect could be absent as hormone breast cancer therapy. Ingle’s study of anastrozole in an approved daily dose of 1 mg revealed significant variability in drug metabolism as well as drug effect. The observed variability suggests that anastrozole as the first choice drug in postmenopausal women with ER-positive BC is the main candidate for pharmacogenomics studies aimed at detecting genetic variation in drug metabolism. The results of these future studies may allow movement towards the goal of truly individualized therapy with anastrozole [5].

A limitation of the study was also the inability to correlate the clinicopathological features of anastrozole treatment’s side effects with the frequency of examined SNPs by DXA. DXA is an overly rough method, which might fail to detect fine changes in bone loss that might be induced by genetic variances of anastrozole metabolizing enzymes. However, in our study group of patients as many as 88% were carriers of CYP3A5*3, the DXA method failed to prove a statistically significant reduction in bone mineral density as a clinicopathological feature of decreased enzyme activity at the molecular level. Therefore, it would be advisable to investigate the markers of bone turnover which might be beneficial to detect increase in bone resorption before evidence of a decrease in bone density has occurred; however, this was beyond the scope of this study. Also, it would be interesting to address the subjective observations of patients in terms of musculoskeletal side effects through a self-reported questionnaire about treatment-related symptoms and health-related quality of life with the frequency of the examined SNPs, similar to that done in the study by Wagner et al., where patient-reported predictors of early treatment discontinuation of anastrozole were explored [44].

Therefore, the clinical significance of CYP3A4*1B, CYP3A5*3 and UGT1A4*2 polymorphisms is yet to be clarified. The interethnic variability of this polymorphism may significantly contribute to drug efficacy and toxicity.

## 5. Conclusions

In conclusion, our study indicated the high prevalence of CYP3A5*3 allele in a Croatian subpopulation of BC patients, which is in line with the frequency of this variant allele in other studies, including European Caucasians. As some studies suggest, a reduced enzyme activity of defective CYP3A5 enzyme could be used in the personalized approach to patients suffering from ER-positive BC in the sense of truly individualized anastrozole therapy. We also demonstrated the strong linkage disequilibrium between CYP3A4*1B and CYP3A5*3 polymorphisms in the same group of subjects. Since CYP3A4, CYP3A5 and UGT1A4 metabolize more than 50% of all prescription drugs, the results of our study may represent the basis for the development of a pharmacogenetics program in Croatia.

## Figures and Tables

**Table 1:** Location and effects of CYP3A4*1B, CYP3A5*3 and UGT1A4*2 polymorphisms.

Polymorphism	Nucleotide Substitution	Reference Single Nucleotide Polymorphism (RS)	Protein Effect and Location	Functional Effect
CYP3A4*1B	−392A > G	rs2740574	Promoter region	Increased transcriptional activity, but clinical effect unclear
CYP3A5*3	698A > G	rs776746	Intron 3	Decreased or lost enzyme activity and expression
UGT1A4*2	70C > A	rs6755571	Exon 1	Decreased glucuronidation activity

**Table 2:** Demographic patient characteristics.

	Median (interquartile range)		*p* *
	Group w/o AIs therapy	AIs treated group	
Age [year]	59.5 (55–68)	65 (59–72)	**0.006**
Height [cm]	163 (158–169.5)	162 (158–165)	0.44
Height in youth [cm]	165 (160–169.7)	164 (160.5–167.8)	0.86
Weight [kg]	71.5 (65.2–79.3)	70 (62.5–80)	0.98
BMI [kg/m^2^]	26.4 (24.4–29.7)	26.9 (24–31.2)	0.60
Age at menopause	50 (46–52)	49 (44–51)	0.16
Age at first menstrual period	13 (12–14)	13 (12–14)	0.76
	Number (%) patients		*p* ^†^
	Group w/o AIs therapy	AIs treated group	
Consumption of dairy products	29 (65.9)	50 (62.5)	0.85
Smoking	6 (13.6)	23 (28.8)	0.08
Alcohol consumption	0	1 (1.3)	>0.99
Vitamin D intake	8 (18.2)	32 (38.8)	**0.03**
Calcium intake	12 (27.3)	24 (30)	0.84
Regular cycles	41 (93.2)	71 (88.8)	0.54
Physical activity (work in the garden, at home)	14 (31.8)	8 (10)	**0.003**
Exercise	36 (81.8)	53 (66.3)	**0.03**
Exercise earlier in the youth	45 (85)	47 (57)	**0.001**

* Mann–Whitney U test; ^†^ Fisher’s exact test. Bold denotes statistical significance.

**Table 3:** Allele and genotype frequencies of CYP3A4, CYP3A5 and UGT1A4 genes in BC patients.

SNP Equilibrium	Therapy Group (n = 82)	W/O Group (n = 44)	Total	** *p*-Value for Hardy–Weinberg Equilibrium
	n (%)	n (%)	n (%)	
**CYP3A4**				
*1A/*1A(A/A)	76 (92.7)	43 (97.7)	119 (94)	0.75
*1A/*1B(A/G)	6 (7.3)	1 (2.3)	7 (6)	
*1B/*1B(G/G)	0 (0)	0 (0)	0 (0)	
**CYP3A5**				
*1/*1(A/A)	0 (0)	0 (0)	0 (0)	0.48
*1/*3(A/G)	11 (13.4)	4 (9.1)	15 (12)	
*3/*3(G/G)	71 (86.6)	40 (90.9)	111 (88)	
**UGT1A4**				
*1/*1(C/C)	59 (95.2)	14 (100)	73 (96.1)	0.86
*1/*2(A/C)	3 (4.8)	0 (0)	3 (3.9)	
*2/*2(A/A)	0 (0)	0 (0)	0 (0)	

** *p*-values express whether Croatian population is similar to respective populations.

**Table 4:** Differences in BMD according to CYP3A4, CYP3A5 and UGT1A4 polymorphisms during treatment with anastrozole.

**CYP3A4**	**wt/wt (n = 74)**	wt/CYP3A4*1B (n = 5)	*p* *
BMD L1–L4	1.02 (0.91–1.15)	1.02 (0.99–1.07)	0.98
BMD total hip	0.89 (0.83–0.97)	1.03 (0.87–1.04)	0.15
BMD femoral neck	0.84 (0.77–0.90)	0.93 (0.76–0.94)	0.95
**CYP3A5**	CYP3A5*3/CYP3A5*3 (n = 70)	wt/CYP3A5*3 (n = 9)	
BMD L1–L4	1.02 (0.91–1.14)	1.07 (0.99–1.15)	0.33
BMD total hip	0.89 (0.84–0.98)	0.91 (0.78–1.0)	0.78
BMD femoral neck	0.84 (0.77–0.90)	0.80 (0.76–0.94)	0.75
**UGT1A4**	**wt/wt (n = 56)**	wt/UGT1A4*2 (n = 3)	
BMD L1–L4	1.03 (0.92–1.15)	1.13 (0.82–1.15)	0.95
BMD total hip	0.91 (0.84–1.0)	0.88 (0.75–1.06)	0.78
BMD femoral neck	0.85 (0.78–0.92)	0.81 (0.75–0.96)	0.70

* Mann–Whitney U test.

**Table 5:** Differences in BMD according to CYP3A4, CYP3A5 and UGT1A4 polymorphisms in the group before anastrozole therapy.

**CYP3A4**	**wt/wt (n = 74)**	wt/CYP3A4*1B (n = 5)	*p* *
BMD L1–L4	1.05 (0.99–1.17)	1.30	-
BMD total hip	0.95 (0.85–1.02)	0.96	-
BMD femoral neck	0.87 (0.81–0.94)	0.87	-
**CYP3A5**	CYP3A5*3/CYP3A5*3 (n = 70)	wt/CYP3A5*3 (n = 9)	
BMD L1–L4	1.05 (0.99–1.17)	1.30 (0.74–1.39)	0.46
BMD total hip	0.95 (0.86–1.01)	0.96 (0.77–1.25)	0.90
BMD femoral neck	0.87 (0.82–0.93)	0.87 (0.71–1.04)	0.86
**UGT1A4**	**wt/wt (n = 56)**	wt/UGT1A4*2 (n = 3)	
BMD L1–L4	1.03 (0.77–1.25)	-	-
BMD total hip	0.95 (0.87–1.01)	-	-
BMD femoral neck	0.85 (0.79–0.94)	-	-

* Mann–Whitney U test.
